# Case Report: Facial-limited eosinophilic annular erythema in a patient with rheumatoid arthritis: successful treatment with tofacitinib

**DOI:** 10.3389/fimmu.2026.1792217

**Published:** 2026-04-20

**Authors:** Man-Ning Wu, Ying Wang, Yan-Ping Bai, Lei Wang

**Affiliations:** 1 Beijing University of Chinese Medicine, Beijing, China; 2 Department of Dermatology, China-Japan Friendship Hospital, National Center for Integrative Medicine, Beijing, China

**Keywords:** eosinophilic annular erythema, facial lesions, JAK inhibitors, rheumatoid arthritis, tofacitinib

## Abstract

We report a case of a 67-year-old man with rheumatoid arthritis (RA) presenting with symmetrical annular plaques confined to the face. Histopathology demonstrated dense dermal eosinophilia without flame figures, consistent with eosinophilic annular erythema (EAE). Unlike the typical truncal distribution, lesions were limited to the face, a presentation reported in approximately 3% of cases. The patient was successfully treated with tofacitinib, a Janus kinase (JAK) inhibitor. This report illustrates a rare facial variant of EAE and supports the efficacy of JAK inhibition in patients with concurrent RA.

## Introduction

EAE is a rare, relapsing dermatosis characterized by urticarial, ring-shaped plaques and tissue eosinophilia (1). It is differentiated from Wells syndrome by the absence of prodromal symptoms and lack of flame figures on histology (2). The pathogenesis is not fully understood but is thought to involve eosinophil-mediated inflammation driven by Th2-related cytokines such as IL-5. Clinically, EAE favors the trunk and extremities; exclusive facial involvement is uncommon, identified in 3.2% of cases in a recent review. The disease typically follows a chronic relapsing course, with lesions recurring over time and variable response to treatment. Additionally, while EAE is associated with various autoimmune conditions, concurrent RA is reported in approximately 6% of patients (3). We present a case of facial-limited EAE in a patient with RA who achieved sustained remission with tofacitinib treatment, providing additional clinical evidence for the strategic use of JAK inhibition in this complex clinical setting.

## Case presentation

A 67-year-old man presented with a four-month history of persistent, symmetrical erythematous plaques confined to the face. The eruption began on the cheeks and gradually expanded, accompanied by mild pruritus. Prior treatment with topical corticosteroids (fluticasone propionate 0.05% cream and dexamethasone acetate) demonstrated limited efficacy. The patient denied photosensitivity. His medical history was significant for a 7-year duration of RA, which had been clinically stable on oral methotrexate (15 mg/week).

**Figure 1:**
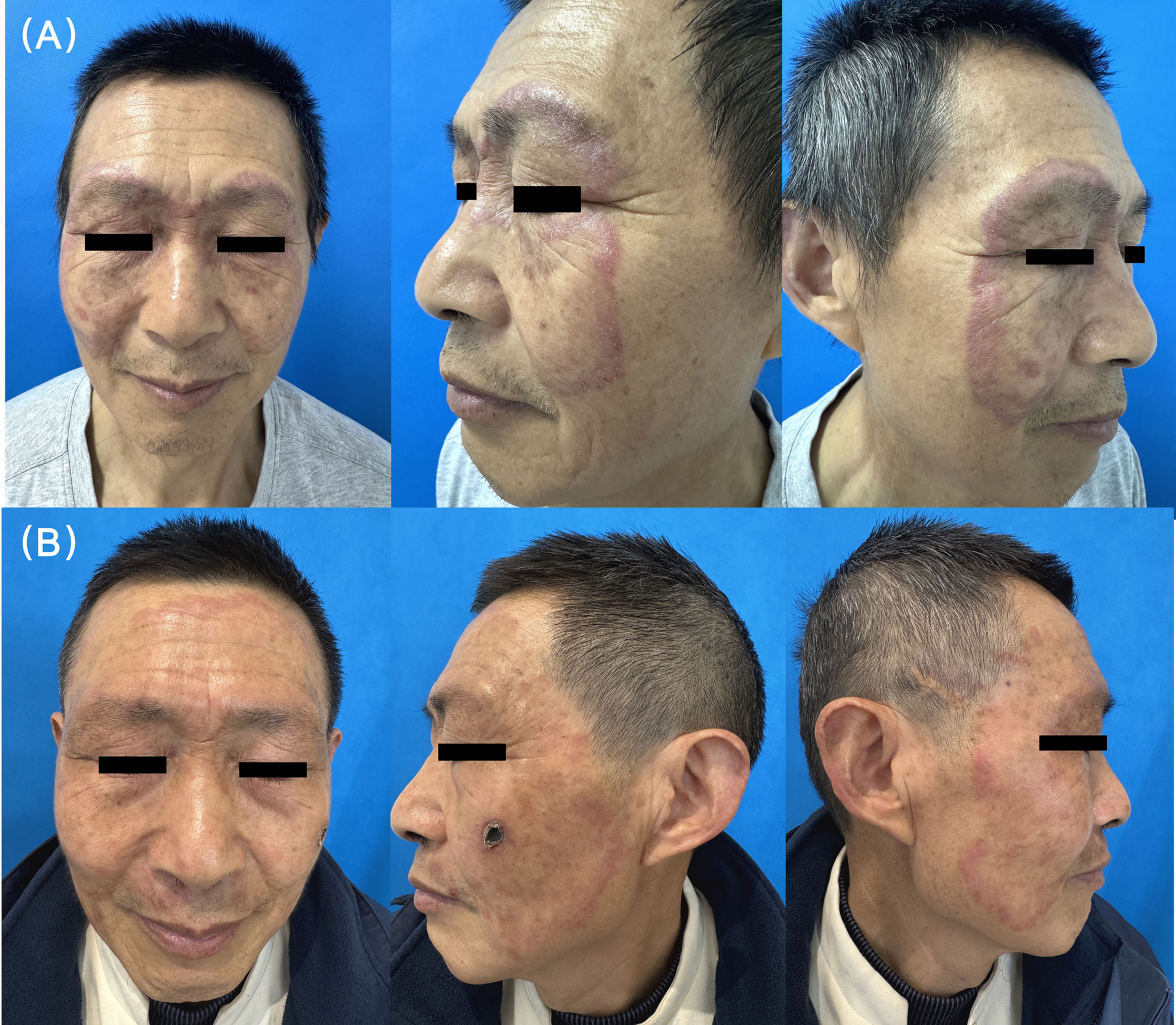
**(A)** Large, symmetrical annular erythematous plaques with violaceous, edematous borders and central clearing. The lesions show centrifugal expansion across the periorbital and mid-facial regions without scaling. **(B)**Significant clinical improvement after treatment with tofacitinib. The previously elevated, erythematous plaques have significantly flattened, with a marked reduction in both edema and the intensity of the erythema, leaving faint residual pinkish patches.

Physical examination revealed multiple well-demarcated, annular, and arcuate erythematous plaques distributed symmetrically across the forehead, periorbital regions, and cheeks. The lesions featured firm, edematous, violaceous borders with mild scaling and central clearing. Atrophy, telangiectasia, and follicular plugging were absent. There were no pustules, vesicles, or mucosal involvement. The trunk and extremities were spared. Regional lymphadenopathy was not palpable (**Figure 1A**).

Laboratory evaluation revealed an elevated rheumatoid factor (101.4 IU/mL), consistent with his history of RA. Complete blood count showed mild monocytosis (0.66 × 10^9^/L) and relative lymphopenia (0.74 × 10^9^/L), with an increased platelet-to-lymphocyte ratio (379.7), suggesting a systemic inflammatory milieu. Serum urea was slightly elevated (10.12 mmol/L), while liver enzymes, complement components (C3, C4), and C1q were within normal limits. The absolute eosinophil count (0.28 × 10^9^/L), erythrocyte sedimentation rate, and C-reactive protein were unremarkable. Autoimmune serologies, including ANA, anti-dsDNA, and anti-ENA antibodies, were negative. Screening for infectious etiologies (syphilis, hepatitis B/C, and HIV) was also negative.

Given the refractory nature of the cutaneous lesions and the concurrent RA, oral tofacitinib citrate 5 mg twice daily was added to his ongoing methotrexate regimen, combined with topical pimecrolimus 1% cream. The patient maintained his baseline methotrexate (MTX) dosage (15 mg/week) for RA management throughout the tofacitinib treatment period, without further dose adjustment. The facial plaques showed significant resolution within one month (**Figure 1B**). The total follow-up duration was 5 months, during which regular outpatient and telephone assessments were conducted. The patient achieved sustained remission, with near-complete resolution of lesions and no recurrence or worsening of joint symptoms. He reported high satisfaction with the treatment, highlighting both symptomatic relief and improved quality of life.

## Histopathological findings

**Figure 2:**
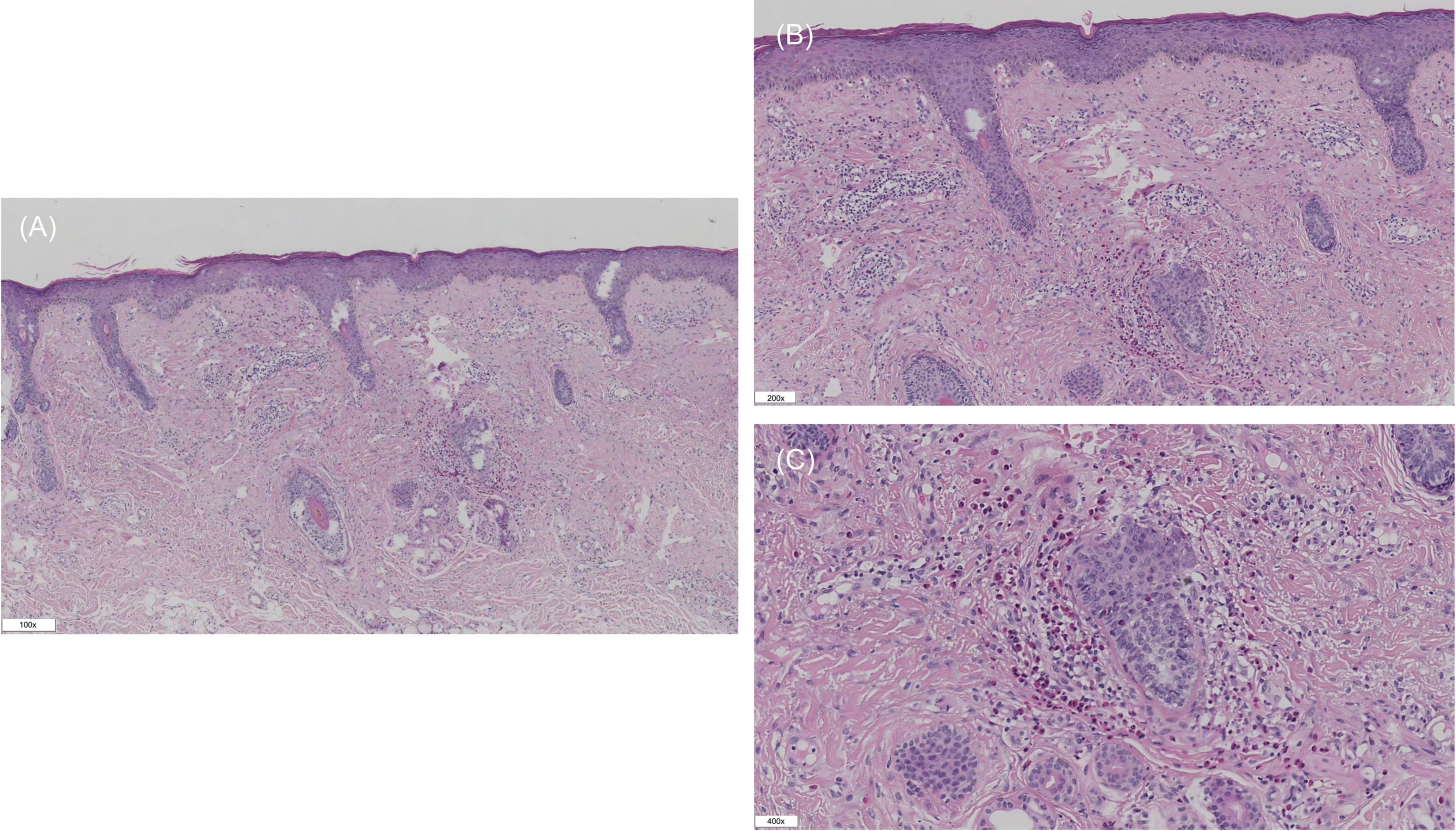
Histopathological evaluation of the facial lesion (H&E stain). **(A)** Low-power view (100×) showing a dense perivascular and periadnexal inflammatory infiltrate in the superficial and mid-dermis. **(B)** Medium-power view (200×) demonstrating that the infiltrate is composed of lymphocytes and numerous eosinophils, accompanied by dermal edema. **(C)** High-power view (400×) highlighting the prominence of eosinophils without evidence of fibrinoid necrosis or flame figures.

Histopathological examination of the facial plaque revealed epidermal hyperkeratosis with focal parakeratosis and mild spongiosis. Interface changes were characterized by basal cell vacuolar alteration and scattered dyskeratotic keratinocytes. In the dermis, there was a dense superficial and mid-dermal inflammatory infiltrate arranged in a perivascular and periadnexal pattern. The infiltrate was composed of lymphocytes, histiocytes, and abundant eosinophils mixed with occasional plasma cells. Dermal edema was present, separating collagen fibers. Importantly, there was no evidence of leukocytoclastic vasculitis, granuloma formation, or the “flame figures” characteristic of Wells syndrome. These findings, correlated with the clinical presentation, supported the diagnosis of eosinophilic annular erythema (**Figure 2**).

## Differential diagnosis

The diagnosis of facial EAE requires careful exclusion of mimics. The primary differential, Wells syndrome, shares tissue eosinophilia but is distinguished by the presence of “flame figures,” which were absent in our case. Given the facial location and RA history, cutaneous lupus erythematosus (CLE) was a critical consideration. However, the absence of interface dermatitis with necrotic keratinocytes (SCLE), dermal mucin (LET), and negative antinuclear antibodies excluded lupus variants. Similarly, granuloma annulare and erythema annulare centrifugum were ruled out based on their distinct histopathological features (palisading granulomas and “coat-sleeve” infiltrates, respectively).

## Discussion

EAE is a rare, relapsing dermatosis characterized by urticarial, ring-shaped plaques and tissue eosinophilia. Current evidence places EAE firmly within the spectrum of Th2-mediated hypersensitivity reactions driven by cytokines such as IL-4, IL-5, and IL-13, which orchestrate eosinophil recruitment and activation (4). Diagnosis requires careful differentiation from mimics. Clinically, while EAE typically favors the trunk, our case presented with exclusive facial involvement. Although Fania et al. (5) suggested the face might be an under-recognized site, a systematic review by Houpe et al. (3) identified exclusive facial involvement in only approximately 3% of cases.

The coexistence of EAE and RA in this patient presents an intriguing immunological paradox. RA is classically driven by Th1 and Th17 pathways, which involve cytokines such as TNF-α, IL-6, and IL-17, whereas EAE represents a distinct Th2-dominant state (6). Literature suggests these opposing pathways may counter-regulate each other. For instance, Bassi et al. (7) reported a case where TNF-α inhibition, a treatment that suppresses the Th1 axis, paradoxically induced EAE in an RA patient. This phenomenon is likely caused by the unmasking of a Th2-skewed immune response. We propose that in patients with long-standing RA, chronic immune dysregulation may establish a state of “cytokine plasticity” that allows local cutaneous Th2 shifts to occur even when systemic Th1 and Th17 pathways are partially controlled (8, 9).

Given this complex dual pathology, JAK inhibitors offer a superior mechanistic rationale over targeted biologics. While the IL-4Rα antagonist dupilumab is effective for refractory EAE, it specifically targets the Th2 axis and lacks efficacy against the Th1 and Th17 drivers of RA (4). In contrast, JAK inhibitors act as a “master key” by broadly modulating the JAK-STAT pathway. Recent clinical evidence has substantiated the efficacy of tofacitinib in EAE; for instance, Niu et al. reported a case of refractory EAE successfully treated with tofacitinib monotherapy, achieving a relapse-free state over an 8-month follow-up (10). Tofacitinib, a JAK1/3 inhibitor, not only blockades the signaling of Th2 cytokines, including IL-4, IL-5, and IL-13, which are essential for EAE pathogenesis—a mechanism supported by the efficacy of other JAK inhibitors like upadacitinib in refractory EAE—but also suppresses the Th1 and Th17 cytokines, such as IL-6 and interferons, that drive the patient’s RA (11).

In our case, the addition of tofacitinib to the patient’s regimen provided a strategic dual-pathway modulation. Despite the relapsing nature characteristic of EAE, our patient achieved near-complete resolution within one month and maintained stable remission throughout a 5-month follow-up period. This favorable outcome should be interpreted in the context of a combination therapeutic approach. Although prior topical corticosteroids were ineffective and EAE is generally difficult to control with topical therapy alone, the introduction of tofacitinib likely played a key role in driving the rapid and sustained clinical response. Importantly, this therapeutic benefit was achieved without adjustment of the baseline MTX regimen, suggesting a synergistic effect between systemic JAK inhibition and concomitant therapy. Taken together, our findings support that combination therapy incorporating tofacitinib may represent an effective strategy for managing EAE in the setting of systemic autoimmune comorbidities, with tofacitinib serving as a central therapeutic component.

## Data Availability

The original contributions presented in the study are included in the article/supplementary material. Further inquiries can be directed to the corresponding author.
